# Left hemisphere predominance of pilocarpine-induced rat epileptiform discharges

**DOI:** 10.1186/1743-0003-6-42

**Published:** 2009-11-30

**Authors:** Yang Xia, Yongxiu Lai, Lei Lei, Yansu Liu, Dezhong Yao

**Affiliations:** 1 Key Laboratory for NeuroInformation of Ministry of Education, School of Life Science and Technology, University of Electronic Science and Technology of China, Chengdu, 610054, China

## Abstract

**Background:**

The left cerebral hemisphere predominance in human focal epilepsy has been observed in a few studies, however, there is no related systematic study in epileptic animal on hemisphere predominance. The main goal of this paper is to observe if the epileptiform discharges (EDs) of Pilocarpine-induced epileptic rats could present difference between left hemisphere and right hemisphere or not.

**Methods:**

The electrocorticogram (ECoG) and electrohippocampogram (EHG) from Pilocarpine-induced epileptic rats were recorded and analyzed using Synchronization likelihood (SL) in order to determine the synchronization relation between different brain regions, then visual check and cross-correlation analysis were adopted to evaluate if the EDs were originated more frequently from the left hemisphere than the right hemisphere.

**Results:**

The data show that the synchronization between left-EHG and right-EHG, left-ECoG and left-EHG, right-ECoG and right-EHG, left-ECoG and right-ECoG, are significantly strengthened after the brain functional state transforms from non-epileptiform discharges to continuous-epileptiform discharges(p < 0.05). When the state transforms from continuous EDs to periodic EDs, the synchronization is significantly weakened between left-ECoG and left-EHG, left-EHG and right-EHG (p < 0.05). Visual check and the time delay (τ) based cross-correlation analysis finds that 10 out of 13 EDs have a left predominance (77%) and 3 out of 13 EDs are right predominance (23%).

**Conclusion:**

The results suggest that the left hemisphere may be more prone to EDs in the Pilocarpine-induced rat epilepsy model and implicate that the left hemisphere might play an important role in epilepsy states transition.

## Background

Functional asymmetry of human brain is a well-known phenomenon at present [1]. Over the last few decades, some literatures reported that focal epileptiform electroencephalography (EEG) patterns may be more likely to occur in the left cerebral hemisphere than in the right [2-5]. Due to asymmetries in anatomic, cytoarchitectonic, developmental, maturation, reorganization and chemical properties between the two hemispheres, some investigators even assert that the left hemisphere is physiologically more predisposed to develop localization-related epilepsy than the right hemisphere [6,7].

Temporal lobe epilepsy (TLE) is the most common drug-resistant type of adult focal epilepsy, which is characterized by hippocampal sclerosis leading to reorganization of neuronal networks. Acute pilocarpine administration, focally in the hippocampus or systemically, leads to limbic seizures in rats with characteristics of human TLE, including similarities in pathology, behavioral abnormalities, as well as occurrence of both partial and generalized seizures [8]. Currently, it is one of the most frequently used ideally models suiting to study the neurobiological mechanisms of epileptogenesis and to test novel compounds for epilepsy treatment [9]. Although there are already some reports on hemisphere preference in human focal epilepsy, there is no related study in Pilocarpine-induced epileptic rat yet. In this study, we analyzed firstly the synchronization relationship of bilateral neocortex and hippocampus epileptiform discharges (EDs) from pilocarpine-induced epileptic rat, then detected the time delay correlation between different brain areas so as to address whether or not the left hemisphere would be more epileptogenic in favour than the right in the epilepsy rat model.

## Methods

### Animals and surgical

This study was conducted on 13 adult male Sprague-Dawley rats weighing 150~250 g obtained from West China Animal Breeding Centre of Sichuan University (China). The breeding and maintenance, as well as all surgical procedures were done under the guidance of Care and Use of Laboratory Animals. The rats were housed individually, kept on a 12 hr on/12 hr off light cycle, controlled room temperature at 22 ± 1 and offered free access to food and water. The animals were allowed to adapt to laboratory conditions for at least 1 week before starting the experiments. All rats were anesthetized with urethane (1 g/kg) and positioned in a stereotaxic apparatus (WPI Stoeling, USA). To monitor electrocorticogram (ECoG) and electrohippocampogram (EHG) changes, two stainless steel screw electrodes were attached to bilaterally neocortex (the electrode placement: 2.5 mm posterior to bregma, 2.5 mm lateral to midline and 0.5 mm above dura) and two Teflon-coated stainless steel wires electrodes (100 μm diameters) with the tip uninsulated were implanted into the dorsal hippocampus (placement of the electrode tips: 3.8 mm posterior to bregma, 2.0 mm lateral to midline and 2.6 mm below dura) according to the Paxinos and Watson Stereotaxic Atlas for Rats [10]. All electrodes were firmly fixed to the skull with dental cement after implantation. A Teflon-coated wire was placed in the rat's ear to serve as the reference electrode and another was placed in beneath the skin of rat's right back limb to serve as the ground electrode. All rats were allowed to recover for three days after the surgery.

All the procedures in this study were approved by the Animal Care Committee of the University of Electronic Science and Technology of China.

### Pilocarpine-induced epileptic discharges and EEG recording

EEG recording was performed under urethane anaesthesia. The EDs was induced by pilocarpine nitrate (Fluka, USA, 380 mg/kg) injected intraperitoneally into all rats. In order to minimize peripheral cholinergic effects, all rats were injected with methylscopolamine (Sigma, USA, 1 mg/kg) 30 min before the application of pilocarpine. About 30 to 40 minutes after pilocarpine treatment, the rats began to appear EDs which was defined as a discharge with frequency higher than 5 Hz and amplitude larger than 2 times of baseline [11].

ECoG from left neocortex(LC), right neocortex(RC) and EHG from left hippocampus(LH), right hippocampus(RH) were obtained using a RM6240C four-channel physiological signal recorder(China). Normal EEG baseline was recorded about 60 minutes before Pilocarpine injection; and then EEG was recorded continuously for another 5 hrs. The EEG epochs were with a sample frequency of 800 Hz and were filtered off-line digitally using a linear 3 order Butterworth filter with a band-pass of 0.5-30 Hz.

### Synchronization likelihood analysis

The Synchronization likelihood (SL) (Appendix A) is a newly developed algorithm for exploring the statistical interdependencies between two or more time series [12,13]. It takes on values between a small number close to 0 in the case of independent time series and 1 in the case of fully synchronized time series. Different from the usual temporal correlation measure, SL is a measure between the reconstructed phase space orbits, thus it is also noted as a chaotic measure.

### Statistical analysis

In this paper, we calculated SL over all possible pairs of channels (LH-RH, LH-LC, RC-RH, LC-RC) to detect which regions are significantly related to the epilepsy states transitions. The results of SL were analyzed by the one-way analysis of variance (ANOVA). The significance level (p-value) was set to 0.05.

### EDs lateralization analysis

To compare the epilepsy sensitivity of different brain regions, visual check and cross-correlation were adopted to analyse EEG between left and right hemisphere. First, all 13 EEG data were determined whether or not EDs have a hemispheric dominance by visual check. Then cross-correlation analysis was adopted to get the time delay (*τ*) for the data which can not be determined by visual check.

Cross-correlation function gives a measure for the correlation or linear synchronization between two time series as a function of time lag *τ *[14]. This function is sensitive to the direction of lag and it may be used to identify the relative time delay of a similar brainwave signal in two simultaneously measured time series [15].

For two discrete univariate EEG time series *x*(*n*) and *y*(*n*) (*n *= 1,..., *N*), the normalized cross-correlation function is defined as

Where *τ *the time lag, *x*(*n*) the reference signal for cross-correlation procedure, *y*(*n*) the signal for evaluation and *w *the window size. Let *τ *be in the range [-*T*, *T*]. The window size *w *and the range of the time shift *T *are very important. In this work, T was in the range from positive 20 ms to negative 20 ms and W was eight sec.

The absolute value of C_*x*,*y*_(*τ*) ranges from 0 (no correlation) to 1 (maximum correlation). We take the lag *τ *at the moment that *C *reaches the maximum value as the time lag *τ *between the two signals. Apparently, the time lag may be positive, negative or equal to 0.

## Results

### EEG feature during status epilepticus induced-pilocarpine

**Figure 1:**
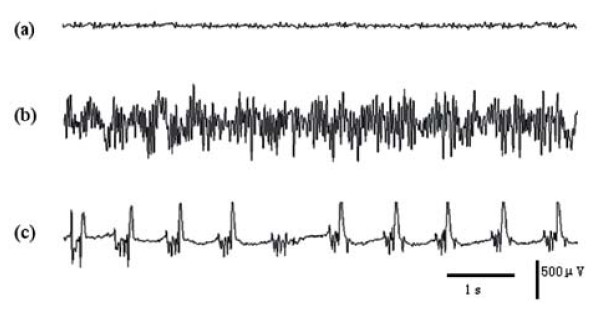
**Epileptiform discharge progression from left neocortex in the pilocarpine epileptic rat model**. (a) Non-EDs. The low amplitude, high frequency activity was firstly observed 1 - 5 min after pilocarpine treatment. (b) Continuous EDs. The prominent high amplitude spiking activity was seen and lasted up to 1~2 hours. (c) Periodic EDs. The bursting spike activity with pause was observed and ended gradually, and resumed to normalize EEG.

As illustrated in Fig. 1, low amplitude, high frequency activity (non-epileptiform discharges) firstly appears in all brain areas recorded, including neocortex and hippocampus, after Pilocarpine treatment (Fig. 1a). After approximately 30 - 40 minutes, high amplitude, low frequency activity replaces the initial low amplitude, high frequency rhythms and shows a discrete seizures activity. Subsequently, an ictal seizure activity is observed, characterized by prominent high amplitude spiking (continuous epileptiform discharges) lasting for 1 - 2 hours (Fig. 1b). During the late stage, EEG is characterized by bursting spike (periodic epileptiform discharges) and gradually resumes to normal EEG (Fig. 1c). This EDs progression was observed in 12 out of 13 rats.

EEG epochs of three different brain functional states, non-epileptiform discharges (non-EDs), continuous epileptiform discharges (continuous EDs) as well as periodic epileptiform discharges (periodic EDs) are selected by visual check for the following analysis.

### SL change along with functional state transition

**Figure 2:**
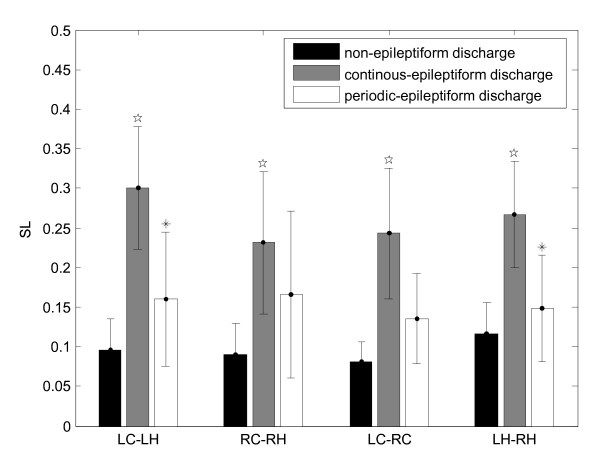
**Mean of Synchronization likelihood index between cerebral regions**. The histogram presents synchronization difference between the two functional states. The pentagram (✩) indicates significant difference from non-EDs to continuous EDs (P < 0.05), and the asterisk (*) indicates significant difference from continuous EDs to periodic EDs (P < 0.05). Values are calculated according to the mean ± SD of the SL index between different brain areas.

We calculated SL for the three functional states to explore the shift between non-EDs, continuous EDs and periodic EDs separately. The results were listed in Table 1 and Fig.2.

In the left column in Table 1, it was found that the synchronizations between left-ECoG and left-EHG, left-EHG and right-EHG were significantly strengthened when the state transformed from non-EDs to continuous EDs (p < 0.05). Similar change also was observed between left-ECoG and right-ECoG, right-ECoG and right-EHG (p < 0.05). When the state transformed from continuous EDs to periodic EDs (as shown in the right column in Table 1), the synchronization was significantly weakened between left-ECoG and left-EHG, as well as between left-EHG and right-EHG (p < 0.05). These data show that left hippocampus (LH) related SL (LH-LC, LH-RH) changed significantly (p < 0.05) when the functional state change occurred and implicate that the left hemisphere might play an important role in epilepsy states transition.

**Table 1:** Synchronization between cerebral regions when different states shifted

	*Form non-epileptiform discharges to continuous-epileptiform discharges*	*Form continuous-epileptiform discharges to periodic-epileptiform discharges*
**LC---LH**	*S_y _non<S_y _continues	*S_y _continues>S_y _periodic
**RC---RH**	*S_y _non<S_y _continues	*S_y _continues = S_y _periodic
**LC---RC**	*S_y _non<S_y _continues	*S_y _continues = S_y_periodic
**LH---RH**	*S_y _non<S_y_continues	*S_y_continues>S_y _periodic

* P < 0.05

### EDs lateralization analysis

**Figure 3:**
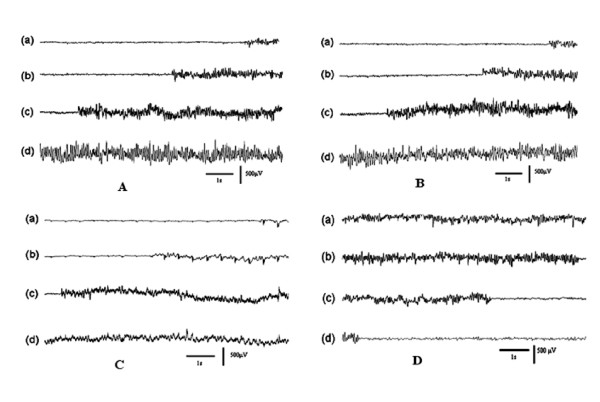
**Epileptiform discharges first onset at left hippocampus three times repeatedly in an epileptic rat**. (A)~(C) illustrate the pattern that EDs onset firstly at left hippocampus 22 sec, 13 sec and 3 sec precedes separately left neocortex in all three times EEG seizures during over recording. (D) illustrates that EDs ending onset from left hippocampus in the same rat. (a) right neocortex, (b) right hippocampus, (c) left neocortex, (d) left hippocampus.

**Figure 4:**
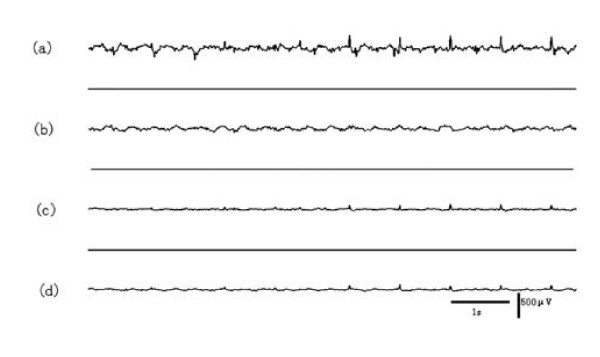
**Epileptiform discharges first appears at right neocortex in another epileptic rat**. (a) right neocortex, (b) right hippocampus, (c) left neocortex, (d) left hippocampus

According to the results of SL, the left hemisphere is crucial for epilepsy states transition. To further confirm whether the EDs activity has lateralization between two hemispheres in this model, we first executed a visual check on all 13 rats EDs, and found that 6 of 13 EDs indicated a visible lateralization with 4 EDs in the left hippocampus and 2 EDs in the right neocortex. In the 6 rats with hemisphere dominance by visual check in our experiment, only 1 rat is observed similar EDs development pattern for three times during the 6 hours of recording. For this pattern, its onset begins at left hippocampus, and then appears at left neocortex, right hippocampus and right neocortex in turn. In Fig.3, it is clear that the epileptiform activity originate from the left hippocampus precedes the other brain areas by 22 s, 13 s, and 3 s separately in the three repeated EDs development pattern observed. The other 5 rats, 3 initiated at left hippocampus and 2 initiated at right neocortex, none shows repeated seizure situation during the 6 hrs of recording. Fig.4 shows a case in a rat that the EDs arise firstly from the right neocortex by visual check, and no repeated seizure situation is observed.

For the 7 rats without lateralization by visual check, we selected a few EEG epochs for each rat during EDs and employed cross-correlation analysis to detect the time delay in order to determine dominance hemisphere.

The greatest correlation coefficient and the related time delay between the two hippocampi were summarized in Table 2. In Table 2, we found 6 of 7 rats showed left predominance (No.1, 2, 3, 4, 5, and 6). One rat showed an EDs lateralization in right hippocampus (No.7). Our results from visual check and the time delay (τ) based correlation analysis show that 10 EDs had a visible left predominance (77%) and 3 EDs were right predominance (23%). These findings suggest that EDs from the left hippocampus has obvious precedence over the right brain areas and demonstrate a distinct left hippocampus predominance.

**Table 2:** Time delay correlation analysis of different brain regions EEG signal

	RH---LH		
		
number	*C*(*X *± *SD*)	τ(*ms*)	dominant side
1	0.8893 ± 0.0780	-3.7500 ± 0.7906	**LH**
2	0.8602 ± 0.0191	-1.7500 ± 0.0847	**LH**
3	0.8753 ± 0.0359	-2.9167 ± 1.7078	**LH**
4	0.5709 ± 0.0736	-17.8125 ± 3.2874	**LH**
5	0.8020 ± 0.0346	-3.5417 ± 0.9410	**LH**
6	0.8300 ± 0.0921	-2.3611 ± 0.7512	**LH**
7	0.7134 ± 0.0784	4.5833 ± 1.0260	**RH**

LH: left hippocampus; RH: right hippocampus; C: the biggest correlation coefficients; τ: the time delay between the two channels signals when they have the biggest C, negative number denotes LH is prior to RH, and vice versa. Data are presented as Mean ± SD.

## Discussion

### Left hemispheres predominance in animal epilepsy

Few papers on hemisphere dominance for epileptic animal model have been published yet, thus a commonly accepted conclusion is still been sought. Although Cain et al. did not observe hemispheric differences in seizure sensitivity and kindling rate in rat model, they noted that most functional and physiological brain asymmetries observed in nonprimate species do not occur consistently in a population. Greater neuronal excitability in the left hemisphere may arise from ontogenetic differences between the two hemispheres that render the left hemisphere more susceptible to cortical damage [16].

In this paper, we studied the epileptic and non-epileptic EEG signals between cortex and hippocampus area for Pilocarpine-induced epileptic rats using SL and cross-correlation. The results proved that left hippocampus (LH) related SL (LH-LC, LH-RH) changes very significantly. Also, the time delay (τ) of electrical activity of different brain areas showed the left hippocampus was more sensitive than the right in Pilocarpine-induced EDs. These findings indicated that the left hippocampus might play an important role during EDs in Pilocarpine-induced rat epilepsy. According to visual check and the time-delay based correlation analysis, our results showed that EDs had a visible left predominance (77%). These preliminary findings raise the possibility that EDs may preferentially originate from the left hippocampus or cortex in our model. We guess that Pilocapine-induced EDs may preferentially originate from left hippocampus or other neighboring brain areas, such as entorhinal cortex, come firstly into left hippocampus, and then spread to other brain regions. This means that the left hippocampus might be more sensitive in seizure than the other brain areas in Pilocarpine-induced epilepsy model.

Although epileptic EEG difference between the two hemispheres is distinct in our study, the true reason has not yet been revealed. However, the lateralization of the seizure onset is an important issue in determining the functional regions of seizure initiation and propagation, and this knowledge of the predominance areas is usually helpful in choosing the appropriate surgical programme clinically. Besides, a more detailed understanding of structural and functional asymmetries in human or animal brain will not only contribute to the identification of the areas for clinic, but can also be meaningful in the evaluation of the cognitive function change before and after a medical treatment.

### Left hemispheres predominance in human epilepsy

Although the details of lateralization of epileptic experimental models are still unclear, this phenomenon in epilepsy patients is already described in early literatures. For instance, Paolozzi et al. observed that two thirds of 4,032 consecutive unselected patients had demonstrated left hemispheric abnormalities [17]. Dean et al. studied the patients in two different laboratories with epileptiform discharge, it was found that spikes of 95 EEG indicating spikes arose from the left in 61 and from the right in 34[18]. Gatzonis et al. reported that 128 of 162 epilepsy patients EEGs showed a strong left predominance (79%) while only 34 patients had a right predominance (23%) [1]. Similarly, left-sided brain tumors seemed much more likely than right-sided tumors to produce seizures. Among craniotomy patients with left hemisphere's tumour, postoperative seizures occurred more frequently with left-sided lesions [19]. Labar et al. discovered that twenty-seven of the patients had lateralized epilepsy: 20 from the left hemisphere and seven from the right hemisphere on 75 epilepsy patients studied using EEG, neuroimaging, ictal semiology and physical examination [20]. Furthermore, Doherty et al. found that the left hemisphere may be more prone to epileptiform discharges in adults, but not to the nonspecific pathophysiologic processes that cause focal EEG slowing [21]. In 2007, Loddenkemper et al. reviewed on 31,207 EEGs (25,793 routine EEGs and 5414 multihour EEGs) recorded during the period from 1993 to 2003. Their result showed that left-sided regional IED were seen in 828 adult patients and accounted for 58% of all unilateral IED, and moreover, there was no lateralization difference in benign focal epileptiform discharges of childhood. So, lateralization shows a tendency toward greater left-sided lateralization of interictal findings with aging [22].

### Physiological basic on left hemispheres predominance

The reason for this EEG discrepancy between the two hemispheres in epilepsy patients or animal model only is speculated. It is widely accepted that the discrepancy between the EEG findings from the two hemispheres should be attributed to their inherent structural and functional organization which leads to the formation of more 'silent' or 'redundant' areas [1].

For human epilepsy, the EDs lateralization may reflect a physiological predisposition for left hemispheric structures to develop focal epilepsy. First, the left hemisphere maturates later than the right, thus remains exposed to harmful agents for longer periods [23,24]. Second, brain anatomy structure and neurochemical organization have differences between the two hemispheres during the nervous system development and differentiation. For instance, postmortem studies have demonstrated asymmetric expression of signal molecules and neurotransmitters, such as γ-aminobutyric acid, dopamine, acetylcholine and their receptors in the human brain [25,26]. This different expression of neurotransmitters and their receptors could lead to different synaptic organization and different epileptic thresholds, consequently lead to differences in epileptogenic susceptibility between the two hemispheres [18]. Besides, carbamazepine has been considered to be an effective antiepileptic agent and may be better in controlling secondarily generalized tonic-clonic seizures from the left side of the EEG focus, suggesting interhemispheric differences in seizure susceptibility [27]. Gur and colleagues found that there were more gray matters relative to white matters and a greater density of cells in the left hemisphere than in the right in human, suggested that the organization of the left hemisphere, relative to that of the right, emphasizes processing or transfer within regions [28].

Beside of the functional and physiological asymmetries observed in human brain, the anatomical brain asymmetries were also found in animals [29]. Specifically, modulating asymmetries of the immune system in the right and left cerebral neocortex have been shown in mice [30]; and some chemical and pharmacologic asymmetries, including those related to catecholamines such as nigrostriatal dopamine content, dopamine receptors, dopamine metabolism have been demonstrated in rats [31]. However, these differences do not give sufficient clues to explain the varied seizure susceptibility between the two hemispheres.

## Conclusion

In conclusion, a notable left lateralization of pilocarpine-induced EDs is observed according to our data (left hippocampus or left cortex). The preliminary findings confirm asymmetric hemispheric functions for focal EDs in animal model and support the hypothesis that the left hemisphere may be more vulnerable to EDs processes.

## Competing interests

The authors declare that they have no competing interests.

## Authors' contributions

YX participated in the design of the experiment carried out the preparation of pilocarpine- induced epileptic rat model and drafted the manuscript. YL carried out the analysis and interpretation of data. LL performed the statistical analysis of data and helped to draft figures and tables of the manuscript. YL participated in acquisition of EEG data and analysis of data. DY was involved in revising the manuscript and gave final approval of the version to be published. All authors read and approved the final manuscript.

## Appendix 1

### Algorithm for synchronization likelihood

We utilized synchronization likelihood (SL) method (Stam,2002; Altenburg,2003) to analyze recorded EEG signal. First, we consider *K *simultaneously recorded time series *x*_*k*,*i*_, where k denotes channel number (*k *= 1, ......, *K*) and *i *denotes discrete time (*i *= 1, ......, *N*). From each of the *K *time series embedded vectors *X*_*k*,*i *_are reconstructed with time-delay embedding:

Where *l *is the lag and *m *is the embedding dimension.

For each time series *k*and each time point *i*, we define the probability  that embedded vectors are closer to each other than a distance *ε*:

Here the |·| is the Euclidean distance and *θ *is the Heaviside step function, *θ*(*x*) = 0 if *x *≤ 0 and *θ*(*x*) = 1 for *x *> 0. Here *w*_1 _and *w*_2 _are widths of two windows; *w*_1 _is the Theiler correction for autocorrelation effects, it should be at least of the order of the autocorrelation time; *w*_2 _is a window that sharpens the time resolution of the synchronization measure, it is chosen such that *w*_1 _<<*w*_2 _<<*N*.

For each *k *and each *i*, the critical distance *ε*_*k*,*i *_is determined for which  = *p*_*ref*_, Where *p*_*ref *_<< 1 is a pre-assumed value.

For each discrete time pair(*i*,*j*) within our considered window (*w*_1_<|*i*-*j*|<*w*_2_), the number of channels *H*_*i*,*j *_where the distance between the embedded vectors *X*_*k*,*i *_and *X*_*k*,*j *_is smaller than the critical distance *ε*_*k*,*i *_is

This number of course lies in a range between 0 and K, and reflects how many of the embedded signals "resemble" each other.

Synchronization likelihood *S*_*k*,*i*,*j *_is defined for each channel k and each discrete time pair(*i*,*j*) as

By averaging over all *j*, with the window (*w*_1_<|*i*-*j*|<*w*_2_), the synchronization likelihood *S*_*k*,*i *_is

In this analysis the following embedding parameters were adopted: lag embedding dimension: *m *= 8; *w*_1 _= 100; *w*_2 _= 200; *p*_*ref *_= 0.05; *N *is the sample number. *S*_y _was obtained by averaging over the time index *i *and channel index *k*.

## References

[B1] Gatzonis SD Roupakiotis S Kambayianni E Politi A Triantafgllou N Mantouvalos V Chioni A Zournas Ch Siafakas A Hemispheric predominance of abnormal findings in electroencephalogram (EEG) Seizure 2002 11 7 442 444 10.1053/seiz.2001.0642 12237070

[B2] Scott D Left and right cerebral hemisphere differences in the occurrence of epilepsy Br J Med Psychol 1985 58 189 192 4016023 10.1111/j.2044-8341.1985.tb02633.x

[B3] Teixeira RA Li LM Santos SL Amorim BJ Etchebehere EC Zanardi VA Guerreiro CA Cendes F Lateralization of epileptiform discharges in patients with epilepsy and precocious destructive brain insults Arq Neuropsiquiatr 2004 62 1 8 15122425 10.1590/s0004-282x2004000100001

[B4] Herzog AG A relationship between particular reproductive endocrine disorders and the laterality of epileptiform discharges in women with epilepsy Neurology 1993 43 10 1907 1910 10.1212/wnl.43.10.1907 8413946

[B5] Holmes MD Dodrill CB Kutsy RL Ojemann GA Miller JW Is the left cerebral hemisphere more prone to epileptogenesis than the right? Epileptic Disord 2001 3 3 137 141 11679305

[B6] Koufen H Gast C Left-sided lateralization and localization off EEG foci in relation to age and diagnosis Arch Psychiatr Nervenkr 1981 229 227 237 7212987

[B7] Kristof M Preiss J Servit J Physiological asymmetry of brain functions-its influence on the lateralization, symptomatology and course of the epileptic process Physiol Bohemoslov 1986 35 447 455 2948206

[B8] Turski L Ikonomidou C Turski WA Bortolotto ZA Cavalheiro EA Review: cholinergic mechanisms and epileptogenesis. The seizures induced by pilocarpine: a novel experimental model of intractable epilepsy Synapse 1989 3 154 171 10.1002/syn.890030207 2648633

[B9] Curia G Longo D Biagini G Jones RSG Avoli M The pilocarpine model of temporal lobe epilepsy Journal of Neuroscience Methods 2008 172 143 157 10.1016/j.jneumeth.2008.04.019 18550176 PMC2518220

[B10] Paxinos G Watson C The Rat Brain in Stereotaxic Coordinates 2005 Elsevier Academic Press, New York

[B11] Goffin K Nissinen J Laere KV Pitkanen A Cyclicity of spontaneous recurrent seizures in pilocarpine model of temporal lobe epilepsy in rat Experimental Neurology 2007 205 501 505 10.1016/j.expneurol.2007.03.008 17442304

[B12] Stam CJ Van Dijk BW Synchronization likelihood: an unbiased measure of generalized synchronization in multivariate data sets Physica D 2002 163 3-4 236 251 10.1016/S0167-2789(01)00386-4

[B13] Altenburg J Vermeulen RJ Strijers RLM Fetter WPF Stam CJ Seizure detection in the neonatal EEG with synchronization likelihood Clinical Neurophysiology 2003 114 1 50 55 10.1016/S1388-2457(02)00322-X 12495763

[B14] Mizuno-Matsumoto Y Okazaki K Kato A Yoshimine T Sato Y Tamura S Hayakawa T Visualization of epileptogenoic phenomena using cross-correlation analysis: localization of epileptic foci and propagation of epileptiform discharges IEEE Trans Bio-Med Eng 1999 46 3 271 279 10.1109/10.748980 10097462

[B15] Oczeretko E Swiatecka J Kitlas A Laudanski T Pierzynski P Visualization of synchronization of the uterine contraction signals: Running cross-correlation and wavelet running cross-correlation methods Medical Engineering & Physics 2006 28 75 81 10.1016/j.medengphy.2005.03.011 15919226

[B16] Cain DP Desborough KA McKitrick DJ Ossenkopp KP Absence of a hemispheric difference in seizure sensitivity and kindling rate in the rat brain Physiol Behav 1989 45 219 20 10.1016/0031-9384(89)90189-3 2727137

[B17] Paolozzi C Hemispheric dominance and asymmetry related to vulnerability of cerebral hemispheres Acta Neurologica 1969 24 13 28 5776652

[B18] Dean A Solomon G Harden S Papakostas G Labar D Left hemispheric dominance of Epileptiform discharges Epilepsia 1997 38 503 505 10.1111/j.1528-1157.1997.tb01743.x 9118859

[B19] Foy PM Chadwick DW Rajgopalan N Johnson AL Shaw MDM Do prophylactic anticonvulsant drugs alter the pattern of seizures after craniotomy? J Neurol Neurosurg Psychiatry 1992 55 753 757 10.1136/jnnp.55.9.753 1402964 PMC1015096

[B20] Labar D Dilone L Solomon G Harden C Epileptogenesis: Left or right hemisphere dominance? Preliminary findings in a hospital-based population Seizure 2001 10 570 572 10.1053/seiz.2001.0565 11792158

[B21] Doherty MJ Walting PJ Morita DC Peterson RA Miller JW Holmes MD Watson NF Do nonspecific focal EEG slowing and epileptiform abnormalities favor one hemisphere? Epilepsia 2002 43 12 1593 1595 10.1046/j.1528-1157.2002.24002.x 12460264

[B22] Loddenkemper T Burgess RC Syed T Pestana EM Lateralization of interictal EEG findings J Clin Neurophysiol 2007 24 5 379 385 10.1097/WNP.0b013e31815607cc 17912060

[B23] Geschwind N Galaburda AM Cerebral lateralization, biologic mechanisms, associations and pathology: I. A hypothesis and a program for research Archives of Neurology 1985 42 428 459 3994562 10.1001/archneur.1985.04060050026008

[B24] Taylor DC Differential rates of cerebral maturation between sexes and between hemispheres Lancet 1969 2 140 142 10.1016/S0140-6736(69)92445-3 4183249

[B25] Amaducci L Sorbi S Albanese A Gainotti G Choline acetyltransfera activity differs in right and left human temporal lobes Neurology 1981 31 799 805 7195501 10.1212/wnl.31.7.799

[B26] Glick SD Ross DA Hough LB Lateral asymmetries of neurotransmitters inhuman brain Brain Res 1982 234 53 63 10.1016/0006-8993(82)90472-3 6120746

[B27] Defazio G Lepore V Specchio LM Pisani F Livrea P The effect of Electroencephalographic focus laterality on efficacy of carbamazepine in complex partial and secondarily generalized tonic-clonic seizures Epilepsia 1991 32 706 711 10.1111/j.1528-1157.1991.tb04713.x 1915180

[B28] Gur R Packer I Hungerbuhler J Differences in the distribution of gray and white matter in human cerebral hemispheres Science 1980 207 1226 1238 10.1126/science.7355287 7355287

[B29] Walker SF Lateralization of functions in the vertebrate brain: A review British Journal of Psychology 1980 71 329 367 7407442 10.1111/j.2044-8295.1980.tb01750.x

[B30] Barneoud P Neveu PJ Vitiello S Moal ML Functional Heterogeneity of the Right and Left Cerebral Neocortex in the Modulation of the Immune System Physiol & Behav 1987 41 525 530 10.1016/0031-9384(87)90306-4 2894692

[B31] Castellano MA Diaz-Palare MD Rodriguez M Barroso J Lateralization in Male Rats and Dopaminergic System: Evidence of Right-Side Population Bias Physiol & Behav 1987 40 607 612 10.1016/0031-9384(87)90105-3 3671525

